# Psychometric Evaluation of the Chinese Version of the Subjective Happiness Scale: Evidence from the Hong Kong FAMILY Cohort

**DOI:** 10.1007/s12529-014-9389-3

**Published:** 2014-02-12

**Authors:** Hairong Nan, Michael Y. Ni, Paul H. Lee, Wilson W. S. Tam, Tai Hing Lam, Gabriel M. Leung, Ian McDowell

**Affiliations:** 1 School of Public Health, The University of Hong Kong, Room 5-05, 5/F, William MW Mong Block, 21 Sassoon Road, Hong Kong, SAR China; 2 School of Nursing, The Hong Kong Polytechnic University, Hong Kong, SAR China; 3 School of Public Health, Chinese University of Hong Kong, Hong Kong, SAR China; 4 Department of Epidemiology and Community Medicine, University of Ottawa, Ottawa, Canada

**Keywords:** Subjective Happiness Scale, Construct validity, Well-being, Family study, Public health, Chinese

## Abstract

**Background:**

With China’s rapid economic growth in the past few decades, there is currently an emerging focus on happiness. Cross-cultural validity studies have indicated that the four-item Subjective Happiness Scale (SHS) has high internal consistency and stable reliability. However, the psychometric characteristics of the SHS in broader Chinese community samples are unknown.

**Purpose:**

We evaluated the factor structure and psychometric properties of the SHS in the Hong Kong general population.

**Methods:**

The Chinese SHS was derived using forward–backward translation. Of the Cantonese-speaking participants aged ≥15 years, 2,635 were randomly selected from the random sample component of the FAMILY Cohort, a territory-wide cohort study in Hong Kong. In addition to the SHS, a single-item overall happiness scale, the Patient Health Questionnaire-9 (PHQ-9), the Family Adaptation, Partnership, Growth, Affection, Resolve (APGAR) scale, and the Medical Outcomes Study 12-item short-form version 2 (SF-12) mental and physical health scales were administered.

**Results:**

Exploratory and confirmatory factor analyses supported a single factor with high loadings for the four SHS items. Multiple group analyses indicated factor invariance across sex and age groups. Cronbach’s alpha was 0.82, and 2-week test–retest reliability (*n* = 191) was 0.70. The SHS correlated significantly with single-item overall happiness (Spearman’s rho [*ρ*] = 0.57), Family APGAR (*ρ* = 0.26), PHQ-9 (*ρ* = −0.34), and mental health-related quality of life (*ρ* = 0.40) but showed a lower correlation with physical health (*ρ* = 0.15). A regression model that included the PHQ-9 and Family APGAR scores explained 37 % of the variance in SF-12 mental health scores; adding the SHS raised the variance explained to 41 %.

**Conclusions:**

Our results support the reliability and validity of the SHS as a relevant component in the measurement battery for mental well-being in a Chinese general population.

## Introduction

As mortality rates decline and people live longer with chronic conditions, attention turns toward enhancing their quality of life, in China as elsewhere. People do not wish merely to survive, but to live productively and happily within the constraints of their condition. To monitor this, attention has turned towards developing measures of positive health states, both objective (such as physical agility and fitness) and subjective (happiness and feelings of well-being). The FAMILY Cohort study was conducted with the project FAMILY: A Jockey Club Initiative for a Harmonious Society. Using baseline data of the cohort [1], we sought to develop positive measures of family health and function, as well as individual well-being and happiness.

There are various conflicting conceptions of happiness. In the West, there is a tension between viewing happiness or feelings of well-being as things that humans desire for their own sake [2] versus the rival eudaimonistic perspective that criticizes hedonic pleasure as being self-indulgent; man should aspire higher than a life of mere pleasure [3]. While Chinese philosophy presents individual happiness as an ideal, the happiness of an individual cannot be achieved without the happiness of others, which is crucial for a harmonious society [4]. Studies during the 1980s focused on predictors of well-being or happiness, such as personality [5], stressful life events [6], economic factors, and social isolation [7]. Later, the focus shifted to an examination of the subjective processes of happiness [8], and Lyubomirsky and Lepper [9] proposed the Subjective Happiness Scale (SHS). This is a four-item scale of subjective happiness, using ratings on 7-point rating scales. Two items cover current state: one rates happiness on an absolute scale and one relative to peers. Two items cover trait happiness via brief descriptions of happy and unhappy individuals, asking participants the extent to which each characterization describes themselves.

The SHS demonstrated Cronbach’s alpha ranging from 0.85 to 0.95, a unitary factor structure, and high test–retest reliability [10]. There is some evidence for its convergent and discriminant validity [11]. Cross-cultural validity of the SHS has been examined in Portuguese [12] and Malay populations [13]. The psychometric properties of the SHS in Chinese community samples are unknown. Using a large random sample from a Cantonese-speaking population in Hong Kong, three research questions were addressed:

1. Do the Chinese SHS scores show adequate psychometric properties?
2. Is there evidence for convergent and discriminant validity?
3. We proposed that the SHS would show incremental validity by explaining unique variance in participants’ well-being as measured by the mental component score of the Medical Outcomes Study 12-item short-form version 2 (SF-12).

## Methods

### Participants and Procedures

A pilot of the FAMILY Cohort, a territory-wide household study, was conducted from February to October 2009. Details of the household survey are described elsewhere [14]. In brief, households were the sampling unit and eligible individuals included Cantonese-speaking family members aged 15 or above. After obtaining written consent, interviewers used laptop computers to administer a questionnaire to each eligible family member. As the objective of the FAMILY Cohort is to study individual- and household-level health, happiness, and family harmony, only households in which all members agreed to participate were included. The sociodemographic characteristics of the study sample were similar with those of the general population of Hong Kong [14]. Since more than one person could be interviewed in each household, and their results would not form independent observations, we randomly selected one household member from each household for the present analyses. A subsample of 191 was randomly selected to complete a re-test of the SHS 2 weeks after the household interview. The study was approved by the Institutional Review Board of the University of Hong Kong/Hospital Authority Hong Kong West Cluster.

### Instruments and Data

Both the four-item SHS and a single-item overall happiness scale were used to measure subjective happiness. The SHS was translated into Chinese with backward translation to English (Appendix). Responses on the 7-point scales were summed and divided by 4 to provide a 1–7 scale with higher scores indicating more happiness [10]. The single-item overall happiness scale [15], used in the World Values Surveys (WVS) [16], asks the participants “All thing considered, would you say you are: very happy (coded 4), happy (coded 3), not very happy (coded 2), or not happy at all? (coded 1)” .

*The Patient Health Questionnaire-9 (PHQ-9)* [17] was derived from the 60-item PHQ [17, 18]. Participants rated how often in the past 2 weeks they had experienced nine symptoms of depression on 4-point frequency scales (0–3). Scores range from 0 to 27, indicating increasingly severe depression symptoms. Scores of 5 or higher indicate mild or higher levels of severity. An internal consistency alpha of 0.91 has been reported [19], while that in the present sample was 0.82 [14].

*The Family Adaptation, Partnership, Growth, Affection, Resolve (APGAR) Questionnaire* collects information on five areas of family function [20]. Three-point frequency answers give an overall range from 0 to 10, indicating improving family function. The Chinese version has shown evidence of reliability and construct validity in Taiwan [21] and Hong Kong [22]. The alpha internal consistency in the present sample was 0.91.

*Physical and Mental Health-Related Quality of Life*. TSF-12 [23] measures eight domains of health on 3- or 5-point answer scales which provides a Physical Component Score (PCS) and a Mental Component Score (MCS). These were converted to norm-based scores (mean, 50; standard deviation, 10) [24]. Higher scores indicate more favorable function [23]. The SF-12 version 2 has been validated in Chinese samples [25, 26]. For our sample, the alpha for the 12-item scale was 0.86. The physical and mental health (PCS and MCS) were negligibly correlated (Spearman *ρ* = −0.15) in the present sample.

Demographic characteristics of the participants were recorded. Socioeconomic status was measured via education (primary, secondary, or tertiary) and monthly family income per head. This was classified into five levels: <2,500, 2,500–5,000, 5,001–10,000, 10,001–20,000, and ≥20,000 Hong Kong dollars (7.8 HK$ = 1 US$). A participant was classified as a full-time worker or student if he/she was currently working or attaining a school/educational institute.

### Statistical Analyses

To examine the factor structure of SHS, the total sample was randomly split into two equal-sized subsamples. Explorative factor analysis (EFA) was used to identify the underlying factors of the SHS. Prior to this analysis, the Kaiser–Meyer–Olkin (KMO) measure of sampling adequacy and the Bartlett’s test of sphericity were examined to evaluate whether the data met the assumptions for carrying out a factor analysis. Factors with an eigenvalue >1 were retained. Next, confirmatory factor analysis (CFA) using package “SEM” [27] of R (R Development Core Team, 2010) was applied to the second subsample to examine the goodness of fit of the observed data to the factor model obtained in the previous step. Model fit was assessed using the standardized root mean square residual (SRMR), the normed fit index (NFI) and the comparative fit index (CFI). An SRMR value of <0.05, and an NFI and CFI of ≥0.95 indicate a good fit [28, 29]. We also examined the invariance of the factor structure in the second subsample, separately by sex and by age group (age <25, 25–44, 45–64, or ≥65).

Homogeneity of factor solution(s) was determined by calculating item–total correlations and internal consistency by Cronbach’s alpha. An alpha of ≥0.7 was regarded as sufficient. Two-week test–retest reliability was assessed by using the one-way random intraclass correlation coefficient. We examined construct validity with the total sample in three domains. First, to assess the convergent validity of the SHS, we computed Spearman rho (*ρ*) correlations with the Family APGAR, the PHQ-9, and the SF-12 PCS and MCS. Second, to assess the discriminant validity, we compared its Spearman correlations with the PCS and the MCS using Fisher’s *z* transformation. We hypothesized that the correlation between the SHS and the MCS would be significantly higher than that with the PCS. We also hypothesized that those with depressive symptoms (PHQ-9 scores ≥5) would be unlikely to score in the top quartile of the SHS. Finally, we examined the incremental contribution of the SHS in predicting mental health by adding the SHS to a multiple regression analysis after including the PHQ-9 and family APGAR. All statistical analyses except CFA were conducted using Predictive Analytics Software for Windows, version 19 (PASW, formerly known as SPSS).

## Results

### Participants

Of the 12,000 addresses randomly drawn from the list of addresses in Hong Kong, 11,106 (92.6 %) were valid. Among the valid addresses, 1,905 were defined as nonresponse after six visits. From the remaining 9,201 addresses, all household members completed the questionnaire in 2,635 households (response rate, 28.8 %). One household member aged 15 or above was randomly selected from each household and formed the analytic sample in the present paper (*N* = 2,635). Their mean age was 49.2 years (SD, 18.8), with a slight preponderance of women (56.4 %) and married participants (57.7 %). Table 1 shows that the overall mean SHS score was 5.07 (SD 1.05). Married people were happier than those divorced or separated (SHS score 5.06 vs. 4.77, *p* < 0.05); those living alone were less happy than those living with other family members (*p* < 0.05). The SHS score increased with income (*p* < 0.01) and those with average income >20,000 HKD reported the highest proportion (32.9 %) in the top quartile. No significant differences were found in scores by sex, age, employment status, and religion.

**Table 1:** Demographic characteristics and Subjective Happiness Scale (SHS) scores of the study sample aged ≥15 years in Hong Kong

Variables	*N* (%)	Mean SHS (SE^a^)	Top quartile of SHS^b^ (%)	Chi-square^b^
Participants	2,635	5.07 (1.05)	593	
Men	1,150 (43.6)	5.05 (1.06)^c^	236 (20.5)	4.63^*^
Women	1,485 (56.4)	5.09 (1.05)	357 (24.0)	
Age group				
15–24	306 (11.6)	5.00 (1.03)^c^	62 (20.3)	14.29^**^
25–44	803 (30.5)	5.02 (1.00)	150 (18.7)	
45–64	933 (35.4)	5.12 (1.01)	224 (24.0)	
65+	593 (22.5)	5.09 (1.18)	157 (26.5)	
Marital status				
Never married	670 (25.5)	4.76 (1.01)	115 (17.2)	22.60^***^
Married	1,517 (57.7)	5.06 (1.01)^*^	375 (24.7)	
Widowed	257 (9.8)	4.80 (1.02)	70 (27.2)	
Divorced/separated	184 (7.0)	4.77 (1.02)^c^	31 (16.8)	
Education levels				
Primary or less	798 (30.5)	4.85 (1.01)^c^	209 (26.2)	9.82^***^
Secondary	1,245 (47.2)	5.00 (1.01)^*^	270 (21.7)	
Tertiary or above	573 (21.7)	4.98 (1.01)	111 (19.4)	
Job status				
Full-time worker	1,120 (42.5)	5.01 (1.01)^c^	244 (21.8)	2.88
Full-time student	215 (8.2)	4.95 (1.02)	41 (19.1)	
Others	1,300 (49.3)	4.88 (1.01)	308 (23.7)	
Persons in household				
1 (living alone)	532 (20.2)	4.74 (1.01)^c^	121 (22.7)	5.26
2	739 (28.0)	4.92 (1.01)^*^	178 (24.1)	
3	603 (22.9)	4.99 (1.01)^*^	128 (21.2)	
4	574 (21.8)	5.06 (1.01)^*^	116 (20.2)	
5+	187 (7.1)	5.05 (1.02)^*^	50 (26.7)	
Level of depressive symptom^d^				
Minimal (0–4)	2,143 (81.3)	5.16 (1.00)^c^	547 (25.5)	69.67^*^
Mild and above (5–27)	492 (18.7)	4.06 (1.01)^***^	46 (9.3)	
Religious affiliation (%)				
No	1,764 (66.9)	4.93 (1.01)^c^	370 (21.0)	7.06^**^
Yes	871 (33.1)	4.94 (1.01)	223 (25.6)	
Household income per head (HK$^e^/month)				
No income or ≤2,500	484 (18.4)	4.84 (1.01)^c^	101 (20.9)	12.22^*^
2,501–5,000	854 (32.4)	4.86 (1.01)	191 (22.4)	
5,001–10,000	643 (24.4)	4.99 (1.01)	130 (20.2)	
10,001–20,000	325 (12.3)	5.08 (1.01)^*^	81 (24.9)	
>20,000	149 (5.7)	5.19 (1.02)^*^	49 (32.9)	

^*^ *p* < 0.05 for the comparison with the reference group and adjusted for sex and age; ^**^ *p* < 0.01; ^***^ *p* < 0.001

^a^SE = standard error with exception of standard deviation for participants, men, women, and age group

^b^Top quartile of the Subjective Happiness Scale (range, 6–7) were classified as “happy” [10]. Chi-square test for top quartile versus other three quartiles

^c^Reference group

^d^Measured by Patient Health Questionnaire-9 (PHQ-9); scores range from 0 to 27, scores ≥5 indicating with mild or above level of depressive symptom

^e^7.8HK$ = 1 US$

### Factor Analysis

The KMO measure (0.76) and Bartlett’s test of sphericity (*p* < 0.001) indicated that the assumptions for factor analysis were met. An EFA yielded a one-factor model with an eigenvalue of 2.64 explaining 65.3 % of the variance; loadings of the four items were 0.87, 0.83, 0.83, and 0.70. The factor structure was the same for men and women, and across age groups. On the second subsample (*n* = 1,309), a CFA showed that the one-factor model identified in the first subsample provided an excellent goodness-of-fit (SRMR, 0.03; NFI, 0.98; and CFI, 0.98).

### Reliability

Based on the complete sample of 2,635 participants, Cronbach’s alpha for the SHS was 0.82, demonstrating good internal consistency, while the corrected item–total correlations were 0.73, 0.67, 0.69 and 0.52 for items 1 through 4, respectively (Table 2). The 2-week test–retest reliability among 191 subjects was 0.70 (*p* < 0.01). The results suggested that item 4 in the SHS might not contribute to the overall theme of the scale in this study sample. The validity analyses that followed accordingly compared the four-item SHS with a three-item abbreviation that omitted the final item.

**Table 2:** Internal consistency of the subjective happiness scale (*N* = 2,635)

Items ^a^	Mean	SD	Item–total correlation	Cronbach’s alpha with item deleted ^b^
1. In general, I consider myself:	4.96	1.21	0.73	0.74
2. Compared to most of my peers, I consider myself:	4.79	1.26	0.67	0.77
3. Some people are generally very happy. They enjoy life regardless of what is going on, getting the most out of everything. To what extent does this describe you?	4.94	1.33	0.69	0.75
4. Some people are generally not very happy. Although they are not depressed, they never seem as happy as they might be. To what extent does this describe you? ^c^	5.59	1.41	0.52	0.84

^a^Response scales use 7-point scales, as shown in the Appendix

^b^Cronbach’s alpha for the complete scale was 0.82

^c^Reverse-coded item

### Convergent and Discriminant Validity

The four-item SHS scores correlated significantly with the single-item happiness scale (*ρ* = 0.57), the Family APGAR (*ρ* = 0.26), the PHQ-9 (*ρ* = −0.34), the SF-12 MCS (*ρ* = 0.40), and PCS (*ρ* = 0.15). Comparison of correlations using Fisher’s *z* transformation revealed that the SHS had a significantly lower correlation with the PCS than with the MCS (*p* < 0.001). SHS scores correlated (*ρ* = 0.26) with the family APGAR, slightly but significantly higher than the equivalent correlation for the single-item happiness scale, at *ρ* = 0.22 (Fisher’s transformation test, *z* = −2.33; *p* = 0.02). The equivalent correlations for the three-item abbreviation were all lower (by *ρ* = 0.01 to 0.03) than those for the four-item scale. These results suggested that the fourth item might measure a different facet of happiness and yet contributed to the overall theme. Participants without depressive symptoms (PHQ-9 scores <5) were happier than those with depression (PHQ-9 score ≥5). Participants with depression were the least likely to score in the top SHS quartile (7.7 %, *p* < 0.001).

### Incremental Validity

Adding the SHS score into a regression model with the MCS as dependent variable and using PHQ-9 and Family APAGR as predictors, increased the variance explained from 37 to 41 % (standardized beta for SHS = 0.22, *p* < 0.001). The SHS score thus contributed additionally to explaining variance in the MCS; the same results were observed using the three- or four-item versions.

Since a few cases lacked data on marital status (7), education (19), and income (180), a sensitivity analysis using those without any missing data (*N* = 2,434) was conducted. The results remained unchanged, the greatest contrast being a reduction of 0.01 in the correlation coefficients of SHS with MCS and PCS.

## Discussion

In this large and representative sample of the Hong Kong population, the Chinese SHS showed consistent factor structure and factorial invariance by sex and age groups, reasonable internal consistency, validity, and test–retest reliability. The one-factor structure of the SHS in our study (eigenvalue 2.64 explaining 65.3 % of the total variance) was virtually identical to the results reported for the German version, with an eigenvalue of 2.63 explaining 65.7 % of the total variance [30]. Our examination of the factor structure using EFA and CFA lends support to the notion that the SHS offers a single-dimensional measure of happiness that holds cross-cultural validity: perhaps Chinese and European notions of happiness are not fundamentally different. The SHS provides a consistent measure of some state or even trait, despite some concern over the fourth item. Our validation study aimed at indicating what this trait might be.

Our study showed a significantly lower correlation between happiness and physical health compared to mental health, consistent with previous studies [31, 32]. In this Chinese sample, subjective happiness added some explanatory power for mental health, suggesting that using the SHS offers more information on subjective well-being than merely depression score and family function. The SHS also performed slightly better than the single-item happiness scale. Family function plays an essential role in Eastern cultures influenced by Confucianism, particularly in times of illness [33] and during treatment of family members [34]. The conception of happiness in Eastern cultures is more collective and linked with the essence of holistic being, completed by achieving through harmony with other individuals, with society, and with nature [4]. Given many cultural similarities shared between Hong Kong and rapidly developing areas in mainland China and other Asian nations, our Hong Kong results may be broadly applicable across Asia.

Limitations of the study included the recruitment of complete households in our sample, which lowered response rates and a potential selection bias towards happier families. To examine this potential bias, we recruited a separate subsample of incomplete households and randomly selected one member from each household (*N* = 150). A comparison showed that happiness scores of these individuals did not substantially differ (Cohen’s *d* effect size = 0.2) from the present sample. Second, participation rate was lower than ideal although our sample was large and covered a wide age range. However, our sample was similar to the Hong Kong census population in sociodemographic characteristics [14]. Third, the study did not include objective medical diagnoses. We have however tested the association between depressive symptoms measured by the PHQ-9, which has good psychometric properties and is a well-established measurement for depressive symptoms in the Chinese populations [14].

In conclusion, our study has provided evidence for the reliability and validity of the Chinese version of the SHS.

## Appendix

Subjective Happiness Scale主觀幸福水平量表

Instructions to participants: “For each of the following statements and/or questions, please circle the point on the scale that you feel is most appropriate in describing you.”

參加者請注意:對於下面的每一個語句和/或問題,請找出你覺得最適當的對你情況的描述,並圈上合適的代表數字
